# Supplementary material for: Deletion Mutants of Francisella Phagosomal Transporters FptA and FptF Are Highly Attenuated for Virulence and Are Protective Against Lethal Intranasal Francisella LVS Challenge in a Murine Model of Respiratory Tularemia
Source: Pathogens. 2021 Jun 24;10(7):799. doi: 10.3390/pathogens10070799 (PMC8308642; doi:10.3390/pathogens10070799)
Supplement: Supplementary file 1 [file pathogens-10-00799-s001.zip › pathogens-1237654-supplementary.pdf]

pathogens

MDPI

Supplementary Materials

Table S1. Attenuation of *fpt* mutant strains in BALB/c mice [1].

Strain	Dose (i.p.) on Day 1 (CFU)	Survival Post-Inoculation on Day 29	LD_{50}
**PBS**	-	4/4; 100%	-
**LVS**	~450	0/4; 0%	<450 CFU
**LVSΔ*fptA***	~3000	4/4; 100%	>3000 CFU
**LVSΔ*fptF***	~6000	4/4; 100%	>6000 CFU

[1] Groups of 4 six- to eight-week-old BALB/c mice were inoculated i.p. with the indicated doses of either LVS, LVSΔ*fptA* or LVSΔ*fptF* strain; or PBS and followed for 29 days post-infection. Mice were euthanized once they lost > 20% of initial starting weight.

Table S2. Scoring criteria for histpathology analysis.

**1) Extent of inflammatory changes**	
**1a) Global extent of Inflammation:** Percentage of surface area	0: <20% of tissue<br>1: >20% and <50%<br>2: >50%
**1b) Surface Area of Alveolar Destruction:** Surface area of alveolar destruction due to the inflammatory infiltrate	0: <20% of tissue<br>1: >20% and <50%<br>2: >50%
**1c) Interstitial Involvement:** Inflammatory infiltrates affecting areas between preserved alveoli	0: <50%<br>1: >50%
**1d) Foci of Inflammation:** Foci of more than 50 inflammatory cells per $4mm^2$ (one diffuse area is considered one focus)	0: <1 per $4mm^2$<br>1: >1 per $4mm^2$
**2) Types of inflammatory cells**	
**2a) Neutrophils**	0: <20% of surface area<br>1: >20%
**2b) Macrophages**	0: <50% of surface area<br>1: >50%
**2c) Lymphocytes**	0: <20% of surface area<br>1: >20%
**3) Other histopathologic features**	
**3a) Edematous exudates and/or fibrin deposition**	0: absent or focal<br>1: present
**3b) Hyperplasia of Type II Pneumocytes:** Only including areas with preserved alveolar architecture	0: <5 out of 10 20x power fields<br>1: >5 out of 10 20x power fields

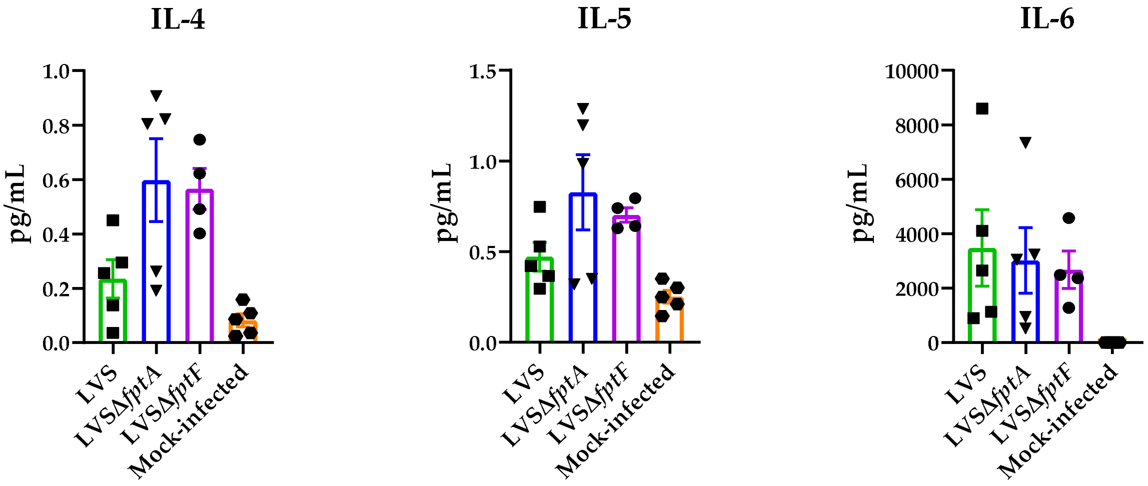

(**A**) (**B**) (**C**)

Figure S1. Cytokine secretion in the bronchoalveolar lavage fluid (BALF) of mice infected with *fpt* mutant strains. Groups of 5 eight-week-old male and female C57BL/6J mice were inoculated i.n. with ~350 CFU of either LVS, LVSΔ*fptA*, LVSΔ*fptF*, or PBS. BALF was harvested at day 6 post-infection for measurement of secreted cytokines using MSD. Symbols indicate cytokine values in individual mice. Bars represent means with SEM from duplicate measurements from one experiment. Levels of IL-4, IL-5 and IL-6 were not significantly elevated in any infection group versus mock infection. One BALF sample from the LVSΔ*fptF* group was excluded from analyses due to inefficient BALF harvest.

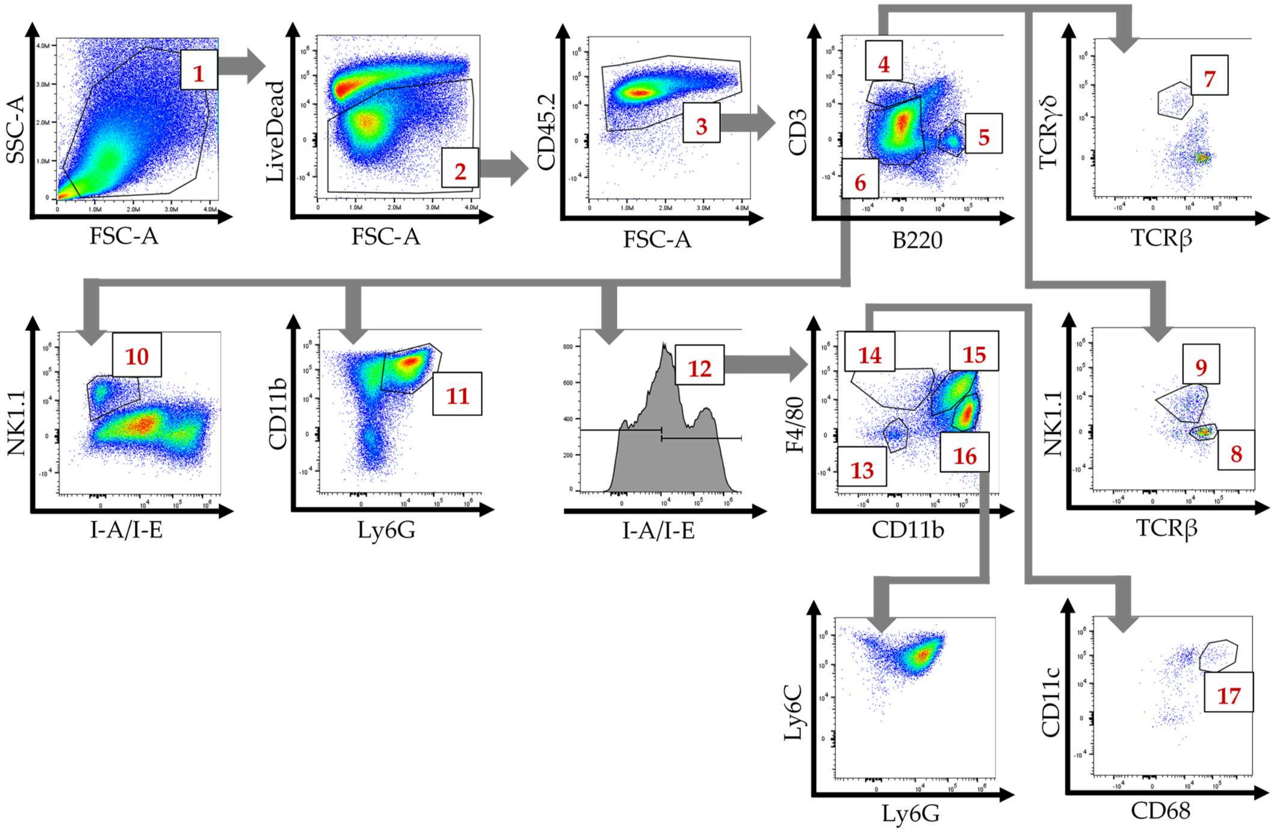

Figure S2. Gating strategy for flow cytometric analysis of infected lung samples. Lungs were harvested and homogenized into single cell suspensions for flow cytometric analysis. Samples were first gated on FSC-A and SSC-A to isolate cells (1). Remaining events were then gated on Zombie NIR− events to identify live cell populations (2). Immune cells were identified by CD45.2+ events (3). These were the events used for the remaining analysis. T cells were defined as CD3+ B220− populations (4). T cells were further subtyped into γδ T cells (γδTCR+TCRβ−, 7), αβ T cells (NK1.1−, TCRβ+, 8), and natural killer-like T (NKT-like) cells (NK1.1+TCRβ+, 9). B cells were defined as CD3− B220+ populations (5). Non-B and T cells were defined as CD3− B220− populations (6). From the non-B and non-T cell population, natural killer (NK) cells were gated as NK1.1+ I-A/I-E− (Class II) (10). Neutrophils were gated from non-B and non-T cell populations as Ly6G+ CD11b+ cells (11). Non-B Class II+ cells were gated from non-B and non-T cell populations as I-A/I-E+ cells (12). From the non-B Class II+ cells, the following populations were defined: dendritic cells (F4/80−, CD11b−, 13), CD11b− macrophages (F4/80+, CD11b−, 14), CD11b+ macrophages (F4/80+, CD11b+, 15), Class II+ neutrophils (F4/80−, CD11b+, 16). The Ly6C and Ly6G expression of the Class II+ neutrophils are shown from population 16. The CD11b− macrophages were further subtyped into alveolar macrophages based on expression of CD68 and CD11c (17). Representative flow plots are shown for an LVS-infected animal. Similar gating strategy was used for all mock-infected and infected animals.

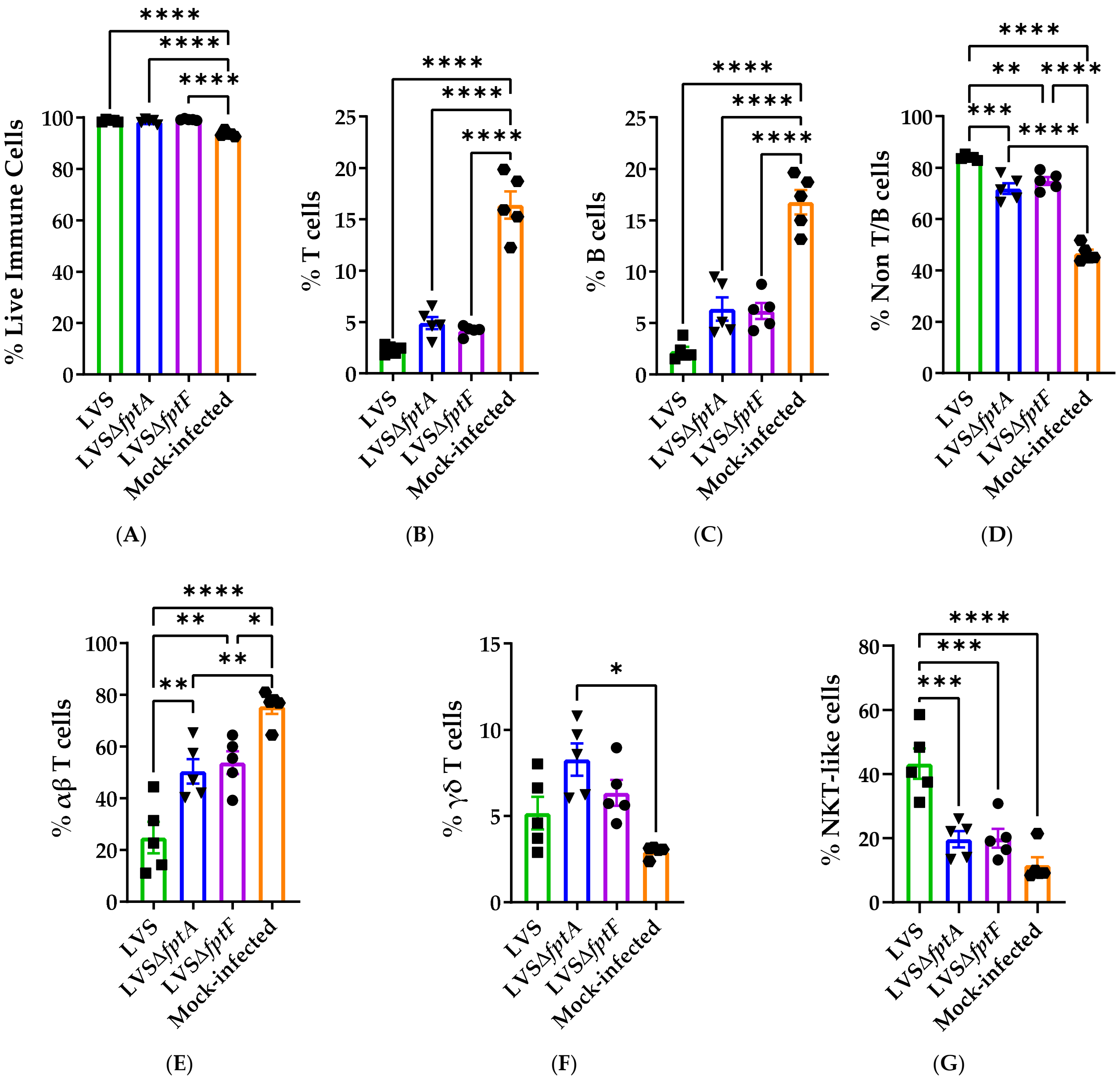

Figure S3. The frequencies of T cells, B cells and non-T/B cells change during infection of mice with *fpt* mutants. Groups of 5 eight-week-old male and female C57BL/6J mice were inoculated i.n. with ~350 CFU of either LVS, LVSΔ*fptA*, or LVSΔ*fptF*, or PBS. Lungs were harvested at day 6 post-infection and stained for flow cytometric analysis of responding immune cell populations. Symbols indicate values from individual mice. Bars represent means with SEM. ****, $p < 0.0001$; ***, $p = 0.001$; **, $p < 0.001$; *, $p < 0.05$ by a one-way ANOVA with a Tukey's post-test. Frequencies of live immune cells (Supplementary Figure S3A), T cells (Supplementary Figure S3B), B cells (Supplementary Figure S3C), non-T/B cells (Supplementary Figure S3D), αβ T cells (Supplementary Figure S3E), γδ T cells (Supplementary Figure S3F), and NKT-like cells (Supplementary Figure S3G) are shown.

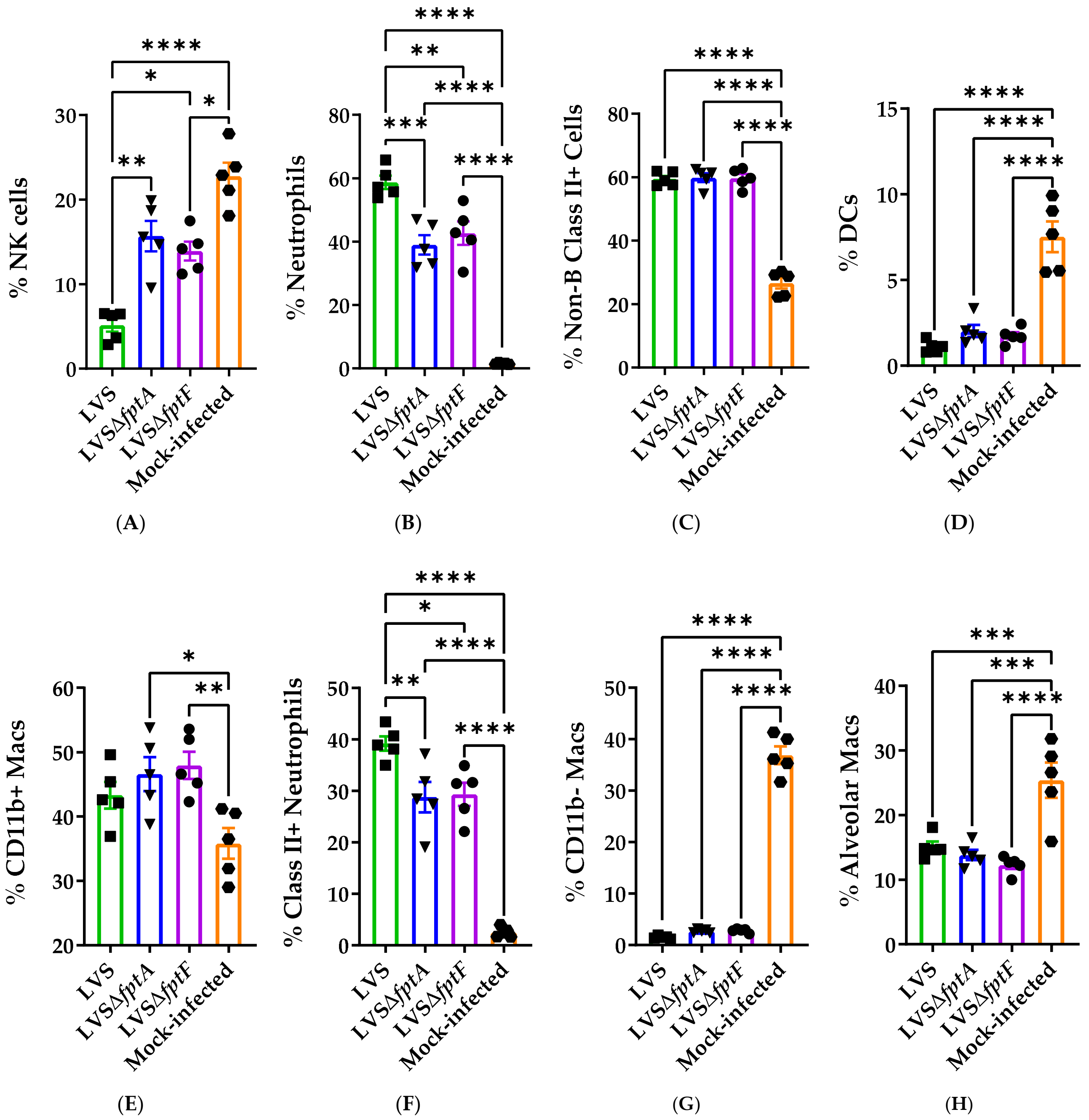

Figure S4. The frequencies of subsets of non-T/B cells change during infection of mice with *fpt* mutants. Groups of 5 eight-week-old male and female C57BL/6J mice were inoculated i.n. with ~350 CFU of either LVS, LVSΔ*fptA*, or LVSΔ*fptF*, or PBS. Lungs were harvested at day 6 post-infection and stained for flow cytometric analysis of responding immune cell populations. Symbols indicate values from individual mice. Bars represent means with SEM. ****, $p < 0.0001$; ***, $p = 0.001$; **, $p < 0.001$; *, $p < 0.05$ by a one-way ANOVA with a Tukey's post-test. Frequencies of NK cells (Supplementary Figure S4A), Neutrophils (Supplementary Figure S4B) non-B Class II+ cells (Supplementary Figure S4C), DCs (Supplementary Figure S4D), CD11b+ macrophages (Supplementary Figure S4E), Class II+ Neutrophils (Supplementary Figure S4F), CD11b− macrophages (Supplementary Figure S4G) and alveolar macrophages (Supplementary Figure S4H) are shown.
